# Sustained virological response to hepatitis C therapy does not decrease the incidence of systemic lupus erythematosus or rheumatoid arthritis

**DOI:** 10.1038/s41598-020-61991-3

**Published:** 2020-03-25

**Authors:** Wei-Fan Hsu, Chi-Yi Chen, Kuo-Chih Tseng, Hsueh-Chou Lai, Hsing-Tao Kuo, Chao-Hung Hung, Shui-Yi Tung, Jing-Houng Wang, Jyh-Jou Chen, Pei-Lun Lee, Rong-Nan Chien, Chun-Yen Lin, Chi-Chieh Yang, Gin-Ho Lo, Chi-Ming Tai, Chih-Wen Lin, Jia-Horng Kao, Chun-Jen Liu, Chen-Hua Liu, Sheng-Lei Yan, Ming-Jong Bair, Wei-Wen Su, Cheng-Hsin Chu, Chih-Jen Chen, Ching-Chu Lo, Pin-Nan Cheng, Yen-Cheng Chiu, Chia-Chi Wang, Jin-Shiung Cheng, Wei-Lun Tsai, Han-Chieh Lin, Yi-Hsiang Huang, Pei-Chien Tsai, Jee-Fu Huang, Chia-Yen Dai, Wan-Long Chuang, Ming-Lung Yu, Cheng-Yuan Peng

**Affiliations:** 1 Center for Digestive Disease, Department of Internal Medicine, China Medical University Hospital, Taichung, Taiwan; 2 Graduate Institute of Biomedical Science, China Medical University, Taichung, Taiwan; 3 Department of Internal Medicine, Chiayi Christian Hospital, Chiayi, Taiwan; 4 Department of Gastroenterology, Dalin Tzu Chi Hospital, Buddhist Tzu Chi Medical Foundation, Chiayi, Taiwan; 5 School of Chinese Medicine, China Medical University, Taichung, Taiwan; 6 Division of Hepato‐gastroenterology, Department of Internal Medicine, Chi-Mei Medical Center, Tainan, Taiwan; 7 Division of Hepatogastroenterology, Department of Internal Medicine, Chiayi Chang Gung Memorial Hospital, Chiayi, Taiwan; 8 Division of Hepatogastroenterology, Department of Internal Medicine, Kaohsiung Chang Gung Memorial Hospital and Chang Gung University College of Medicine, Kaohsiung, Taiwan; 9 Division of Gastroenterology and Hepatology, Chi-Mei Medical Center, Liouying, Tainan Taiwan; 10 Division of Hepatology, Department of Gastroenterology and Hepatology, Linkou Chang Gung Memorial Hospital, Taoyuan, Taiwan; 11 Division of Gastroenterology, Department of Internal Medicine, Show-Chwan Memorial Hospital, Changhua, Taiwan; 12 Division of Gastroenterology and Hepatology, Department of Medicine, E-Da Hospital, I-Shou University, Kaohsiung, Taiwan; 13 Graduate Institute of Clinical Medicine, National Taiwan University College of Medicine, Taipei, Taiwan; 14 Division of Gastroenterology and Hepatology, the National Taiwan University Hospital, Taipei, Taiwan; 15 Division of Gastroenterology, Department of Internal Medicine, Chang Bing Show-Chwan Memorial Hospital, Changhua, Taiwan; 16 Division of Gastroenterology, Department of Internal Medicine, Taitung Mackay Memorial Hospital, Taitung, Taiwan; 17 Division of Gastroenterology, Department of Internal Medicine, Changhua Christian Hospital, Changhua, Taiwan; 18 Division of Gastroenterology, Department of Internal Medicine, Mackay Memorial Hospital, Taipei, Taiwan; 19 Department of Internal Medicine, St. Martin De Porres Hospital - Daya, Chiayi, Taiwan; 20 Division of Gastroenterology and Hepatology, Department of Internal Medicine, National Cheng Kung University Hospital; College of Medicine, National Cheng Kung University, Tainan, Taiwan; 21 Division of Gastroenterology, Department of Internal Medicine, Taipei Tzuchi Hospital, New Taipei City, Taiwan; 22 Division of Gastroenterology and Hepatology, Department of Medicine, Kaohsiung Veterans General Hospital, Kaohsiung, Taiwan; 23 Division of Gastroenterology and Hepatology, Department of Medicine, Taipei Veterans General Hospital, Taipei, Taiwan; 24 Hepatobiliary Division, Department of Internal Medicine and Hepatitis Center Kaohsiung Medical University Hospital, Kaohsiung Medical University, Kaohsiung, Taiwan; 25 School of Medicine and Hepatitis Research Center, College of Medicine, and Cohort Research Center, Kaohsiung Medical University, Kaohsiung, Taiwan; 26 School of Medicine, China Medical University, Taichung, Taiwan

**Keywords:** Hepatitis C, Acute kidney injury, Chronic kidney disease

## Abstract

In patients with chronic hepatitis C (CHC), the effects of baseline characteristics, virological profiles, and therapeutic outcome to pegylated interferon plus ribavirin (PR) therapy on autoimmune diseases are unknown. Taiwanese Chronic Hepatitis C Cohort is a nationwide hepatitis C virus registry cohort comprising 23 hospitals of Taiwan. A total of 12,770 CHC patients receiving PR therapy for at least 4 weeks between January 2003 and December 2015 were enrolled and their data were linked to the Taiwan National Health Insurance Research Database for studying the development of 10 autoimmune diseases. The mean follow-up duration was 5.3 ± 2.9 years with a total of 67,930 person-years, and the annual incidence of systemic lupus erythematosus (SLE) or rheumatoid arthritis (RA) was 0.03%. Other autoimmune diseases were not assessable due to few events. Body mass index ≥24 kg/m^2^ was an independent predictor of the low incidence of SLE or RA (hazard ratio 0.40, 95% confidence interval 0.17–0.93, p = 0.034). A sustained virological response (SVR) to PR therapy was not associated with the low incidence of SLE or RA in any subgroup analysis. CHC patients achieving SVR to PR therapy did not exhibit an impact on the incidence of SLE or RA compared with non-SVR patients.

## Introduction

Chronic hepatitis C (CHC) is well known for its hepatitis C virus (HCV)-associated systemic diseases with extrahepatic manifestations (EMs), such as diabetes mellitus (DM), mixed cryoglobulinemia (MC), systemic lupus erythematosus (SLE), rheumatoid arthritis (RA), Sjögren’s syndrome (SS), and porphyria cutanea tarda^1^. MC vasculitis increases HCV-related morbidity and mortality^2^. Approximately 40–74% of CHC patients experience immunological complications during the course of the disease^2^. The awareness of these immunological complications of HCV infection facilitates hepatologists to diagnose these patients early^3^. On the other hand, patients with rheumatic diseases are often tested for HCV infection^1^.

A previous Taiwan National Health Insurance Research Database (NHIRD) study reported that antiviral treatment with pegylated interferon plus ribavirin (PR) does not decrease the incidence of catastrophic autoimmune diseases^4^, but the effects of baseline characteristics, virological profiles, and therapeutic outcome on autoimmune diseases are unknown. Although direct-acting antiviral agents (DAAs) are the standard of care for CHC^6,6^, the data of the effect of a sustained virological response (SVR) to PR therapy on the incidence of autoimmune diseases provide valuable information for physicians.

We conducted this nationwide cohort study to elucidate the effects of baseline factors and therapeutic outcome of PR therapy on the incidence of autoimmune diseases in CHC patients.

## Results

### Baseline characteristics of the study population

**Figure 1:**
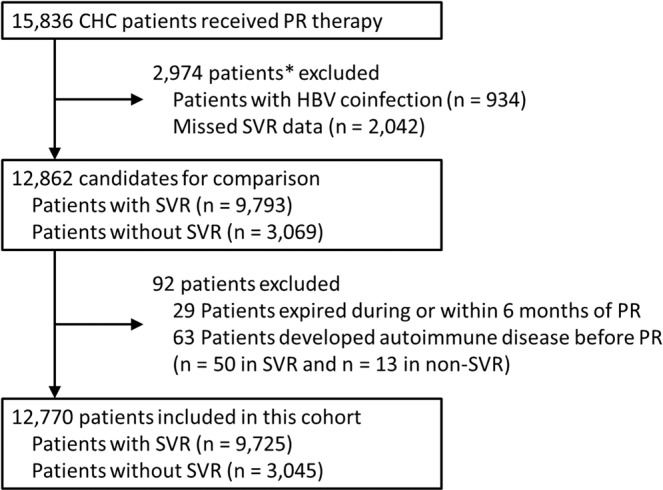
Flowchart of patients enrolled in this study. *2 patients had both HBV infection and unavailable virological outcomes.

A total of 15,836 patients were enrolled initially. Among them, 934 and 2,042 patients were excluded because of hepatitis B virus (HBV) coinfection and unavailable SVR status, respectively (2 patients had both); 29 patients were excluded because of death during or within 6 months of PR therapy, and 63 patients were excluded because of SLE or RA development before PR therapy. Finally, 12,770 patients (9,725 patients with SVR and 3,045 patients without SVR) were analyzed (Fig. 1).

The mean age was 54.6 ± 11.4 years, and 5,954 (46.6%) patients were men. The mean body mass index (BMI) was 25.0 ± 3.5 kg/m^2^. The baseline mean aspartate aminotransferase (AST) and alanine aminotransferase (ALT) levels were 91.1 ± 64.4 and 137.4 ± 110.3 U/L, respectively. The mean platelet count was 174.7 ± 54.0 × 10^9^/L, and mean FIB-4 was 2.9 ± 2.5. Furthermore, 1,969 (18.5%) patients had liver cirrhosis (LC), and 1,269 (18.2%) and 1,411 (20.3%) patients had DM and hypertension, respectively. In total, 6,052 (47.4%), 5,811 (45.5%), and 907 (7.1%) patients were diagnosed with HCV genotypes 1, 2, and non-1/2, respectively, and the median HCV RNA level was 5.7 ± 1.0 log_10_ IU/mL. The mean PR therapy time was 7.1 ± 2.9 months. The mean follow-up duration was 5.3 ± 2.9 years with a total of 67,930 person-years. The SVR rate at 24 weeks after PR therapy was 76.2%. The annual incidence of SLE or RA was 0.03% (Table 1).

**Table 1:** Patient demographics and baseline characteristics.

Variable	Total (*n* = 12,770)	SVR (*n* = 9,725)	Non-SVR (*n* = 3,045)	p value
Age (years)	54.6 ± 11.4	53.9 ± 11.5	56.8 ± 10.7	<0.001
Age ≤55/>55, *n* (%)	6,270 (49.1)/6,500 (50.9)	5,047 (51.8)/4,668 (48.2)	1,233 (40.5)/1,812 (59.5)	<0.001
Sex, M/F (% male)	5,954/6,816 (46.6)	4,379/5,346 (45.0)	1,575/1,470 (51.7)	<0.001
BMI (kg/m^2^)	25.0 ± 3.5	24.9 ± 3.5	25.3 ± 3.6	<0.001
BMI < 24/≥24, *n* (%)	4,368 (34.2)/8,402 (65.8)	3,402 (35.0)/6,323 (65.0)	966 (31.7)/2,079 (68.3)	<0.001
Person-years	67,930	52,754	15,176	
Follow-up duration (years)	5.3 ± 2.9	5.4 ± 3.0	5.0 ± 2.8	<0.001
Platelet count (X 10^9^/L)	174.7 ± 54.0	177.1 ± 51.6	166.9 ± 60.3	<0.001
AST (U/L)	91.1 ± 64.4	91.7 ± 65.9	89.0 ± 59.3	0.037
ALT (U/L)	137.4 ± 110.3	142.6 ± 115.3	120.8 ± 90.9	<0.001
Creatinine (mg/dL)	1.0 ± 1.0	1.0 ± 0.9	1.0 ± 1.2	0.069
eGFR (mL/min/1.73m^2^)	99.6 ± 34.9	99.4 ± 34.8	100.1 ± 35.3	0.356
Liver cirrhosis: no/yes, *n* (%)	8,687 (81.5)/1,969 (18.5)	6,870 (84.7)/1,243 (15.3)	1,817 (23.0)/726 (77.0)	<0.001
HCV RNA (log_10_ IU/mL)	5.7 ± 1.0	5.6 ± 1.0	6.0 ± 0.8	<0.001
HCV RNA ≤ 400,000/>400,000 (IU/mL), *n* (%)	4,379 (39.1)/6,826 (60.9)	3,773 (44.0)/4,796 (56.0)	606 (23.0)/2,030 (77.0)	<0.001
PR therapy time	7.1 ± 2.9	6.9 ± 2.8	7.5 ± 3.3	<0.001
Rapid virological response, yes/no	6,111 (62.5)/3660 (37.5)	5,618 (72.5)/2135 (27.5)	493 (24.4)/1525 (75.6)	<0.001
Virological response at EOT				
Null response	952 (10.3)	None	952 (43.0)	<0.001
Partial virological response	110 (1.2)	None	110 (5.0)	<0.001
Relapse	1,151 (12.4)	None	1,151 (52.0)	<0.001
Genotype, *n* (%)				
1	6,052 (47.4)	4,082 (42.0)	1,970 (64.7)	<0.001
2	5,811 (45.5)	4,940 (50.8)	871 (28.6)	
Non-1/2	907 (7.1)	703 (7.2)	204 (6.7)	
FIB-4	2.9 ± 2.5	2.8 ± 2.3	3.4 ± 3.0	<0.001
FIB-4 < 3.25/≥3.25, *n* (%)	9,063 (71.0)/3,707 (29.0)	7,115 (73.2)/2,610 (26.8)	1,948 (64.0)/1,097 (36.0)	<0.001
DM: no/yes, *n* (%)	5,698 (81.8)/1,269 (18.2)	4,339 (80.0)/904 (20.0)	1,359 (79.0)/365 (21.0)	<0.001
Hypertension: no/yes, *n* (%)	5,556 (79.7)/1,411 (20.3)	4,194 (80.0)/1,049 (20.0)	1,362 (79.0)/362 (21.0)	0.375
Annual incidence of autoimmune disease, *n* (%)	21 (0.03)	15 (0.03)	6 (0.04)	0.799
Annual incidence of SLE, *n* (%)	5 (0.007)	3 (0.006)	2 (0.013)	0.343
Annual incidence of RA, *n* (%)	16 (0.024)	12 (0.023)	4 (0.026)	1.000
Death before autoimmune disease or last visit	653 (5.11)	334 (3.43)	319 (10.48)	0.013

ALT, alanine aminotransferase; AST, aspartate aminotransferase; BMI, body mass index; DM, diabetes mellitus; eGFR, estimated glomerular filtration rate; EOT, end of pegylated interferon plus ribavirin therapy; F, female; HCV, hepatitis C virus; M, male; PR, pegylated interferon plus ribavirin; RA, rheumatoid arthritis; SLE, systemic lupus erythematosus; SVR, sustained virological response.

### Cumulative incidence rate of SLE or RA

The mean age of the patients who achieved SVR to PR therapy (SVR group) was 53.9 ± 11.5 years and of those who did not achieve SVR (non-SVR group) was 56.8 ± 10.7 years (p < 0.001). The median follow-up periods for the SVR and non-SVR groups were 5.4 ± 3.0 and 5.0 ± 2.8 years (p < 0.001), respectively. The SVR group exhibited lower BMI, HCV RNA levels, and FIB-4 index; lower proportion of LC; higher platelet count, and AST and ALT levels; shorter PR therapy time; and higher rate of rapid virological response. The SVR group exhibited a higher proportion of HCV genotype 2 infection than did the non-SVR group (50.8% vs 28.6%, p < 0.001). The SVR group exhibited a lower rate of death before developing autoimmune diseases or last visit than did the non-SVR group (3.43% vs 10.48%, p = 0.013). In the non-SVR group, 43.0, 5.0, and 52.0% of patients had a decline in HCV RNA of ≤2 log_10_ IU/mL (null response), a decline in HCV RNA of >2 log_10_ IU/mL but with detectable HCV RNA (partial virological response), and undetectable HCV RNA at the end of PR therapy (relapse), respectively.

**Figure 2:**
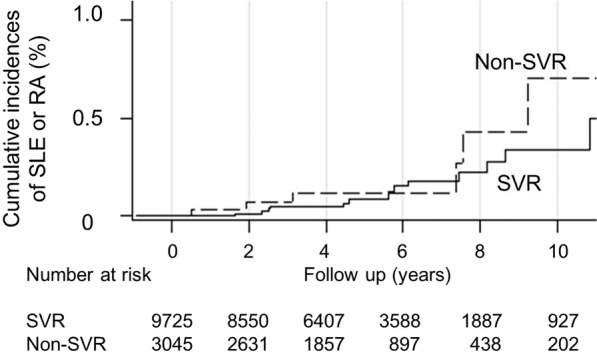
Cumulative incidence of SLE or RA in SVR and non-SVR groups determined using the modified log-rank test after adjustment for age, sex, and competing mortality. SVR patients achieved a sustained virological response (SVR) to pegylated interferon plus ribavirin (PR) therapy; non-SVR patients did not achieve SVR to PR therapy.

The annual incidence of SLE, RA, and SLE or RA did not differ between the SVR and non-SVR groups (0.006% vs. 0.013%, p = 0.343; 0.023% vs. 0.026%, p = 1.000; 0.03% vs 0.04%, p = 0.799) (Table 1). The 10-year cumulative incidence rates of SLE or RA, estimated using the modified Kaplan–Meier method and Gray’s method, were 0.34% (95% confidence interval [CI] 0.17–0.62) for the SVR group and 0.71% (95% CI 0.23–1.75) for the non-SVR group (p = 0.580) (Fig. 2).

### Relative risks of SLE or RA after adjustment for competing mortality

Table 2 shows the multivariate Cox proportional hazard analysis for determining the independent predictors of the SLE or RA. The non-SVR group did not exhibit an increased risk for SLE or RA compared with the SVR group (Hazard ratio [HR] 1.32, 95% CI 0.50–3.51; p = 0.580) after adjustment for age, sex, and competing mortality (Fig. 2). Few patients with hypertension or an estimated glomerular filtration rate (eGFR) of <60 mL/min/1.73m^2^ exhibited SLE or RA (<3 events); therefore, these two factors were not listed for comparison. BMI ≥ 24 kg/m^2^ was an independent predictor of the low incidence of SLE or RA (HR 0.40, 95% CI 0.17–0.93, p = 0.034) after adjustment for competing risk. Other factors, such as age, ALT levels, HCV RNA levels, and LC, did not exhibit significant differences.

**Table 2:** Cox proportional hazards models for risk of SLE or RA after adjustment for competing mortality.

Variable	HR (95% CI)	p value
Non-SVR	1.38 (0.54–3.56)	0.506
Age (years) ≥55 vs <55	0.89 (0.36–2.15)	0.787
Female: male	2.40 (0.98–5.93)	0.056
BMI (kg/m^2^) ≥24 vs <24	0.40 (0.17–0.93)	0.034
AST (U/L) ≥80 vs <80	1.07 (0.46–2.50)	0.885
ALT (U/L)≥80 vs <80	0.80 (0.30–2.14)	0.661
Liver cirrhosis	1.21 (0.40–3.66)	0.731
HCV RNA (IU/mL) >400,000 vs ≤400,000	1.31 (0.47–3.65)	0.613
HCV genotype 2 vs 1	1.83 (0.63–5.34)	0.270
FIB-4 ≥ 3.25 vs <3.25	0.92 (0.36–2.36)	0.869
Diabetes mellitus	1.30 (0.36–4.64)	0.690

ALT, alanine aminotransferase; AST, aspartate aminotransferase; BMI, body mass index; CI, confidence interval; eGFR, estimated glomerular filtration rate; HCV, hepatitis C virus; HR, hazard ratio; SVR, sustained virological response.

### Effect of SVR on the incidence of SLE or RA in different subgroups

Table 3 presents the effect of SVR on the incidence of SLE or RA in different subgroups. SVR to PR therapy was not associated with the low incidence of SLE or RA in any subgroup analysis, stratified by age, sex, BMI, AST and ALT levels, eGFR, HCV RNA levels, HCV genotype, FIB-4 index, DM, hypertension, and LC (all p > 0.05).

**Table 3:** Effect of SVR on the incidence of SLE or RA in different subgroups.

Variable	Conditions	Non-SVR (%)	SVR (%)	Crude HR (95% CI)	p value
Age (years)	<55	0.16	0.32	2.25 (0.69–7.36)	0.182
	≥55	0.15	0.11	0.73 (0.15–3.63)	0.700
Sex	Male	0.07	0.20	3.01 (0.68–13.39)	0.149
	Female	0.25	0.19	0.82 (0.23–2.93)	0.763
BMI (kg/m^2^)	<24	0.24	0.41	1.88 (0.56–6.34)	0.307
	≥24	0.11	0.10	0.91 (0.19–4.41)	0.907
AST (U/L)	<80	0.15	0.12	0.89 (0.19–4.22)	0.883
	≥80	0.16	0.30	1.85 (0.54–6.35)	0.326
ALT (U/L)	<80	0.16	0.09	0.64 (0.07–5.61)	0.686
	≥80	0.15	0.26	1.79 (0.61–5.23)	0.291
eGFR (mL/min/1.73m^2^)	≥60	0.15	0.21	1.49 (0.57–3.89)	0.412
	<60	0.25	0	NA	NA
Liver cirrhosis	No	0.17	0.17	1.10 (0.31–3.90)	0.885
	Yes	0.24	0.14	0.61 (0.06–6.16)	0.675
HCV RNA (IU/mL)	≤400,000	0.16	0	NA	NA
	>400,000	0.10	0.20	2.07 (0.53–7.99)	0.293
HCV genotype	1	0.07	0.15	2.30 (0.46–11.45)	0.309
	2	0.22	0.23	1.26 (0.28–5.67)	0.765
FIB-4	<3.25	0.15	0.21	1.45 (0.47–4.53)	0.521
	≥3.25	0.15	0.18	1.31 (0.23–7.49)	0.760
DM	No	0.16	0.29	2.38 (0.66–8.51)	0.183
	Yes	0.33	0	NA	NA
Hypertension	No	0.21	0.29	1.78 (0.53–5.97)	0.350
	Yes	0.10	0	NA	NA

ALT, alanine aminotransferase; AST, aspartate aminotransferase; BMI, body mass index; DM, diabetes mellitus; eGFR, estimated glomerular filtration rate; HCV, hepatitis C virus; NA, not applicable; RA, rheumatoid arthritis; SLE, systemic lupus erythematosus; SVR, sustained virological response.

## Discussion

In this multicenter retrospective study, we observed that SVR to PR therapy does not decrease the incidence of SLE or RA in CHC patients. BMI ≥ 24 kg/m^2^ was the only independent predictor of the low incidence of SLE or RA.

Taiwanese Chronic Hepatitis C Cohort (T-COACH) is a nationwide HCV registry consortium that includes 23 regional hospitals and medical centers of Taiwan from January 2003 to December 2015, and T-COACH accounted for 21% of the treated CHC population of Taiwan over the 13-year period. In addition to International Classification of Disease, ninth revision, Clinical Modification (ICD-9-CM), T-COACH included baseline characteristics, virological profiles, and therapeutic outcomes. In this study, we enrolled 12,770 patients (9,725 patients with SVR and 3,045 patients without SVR) for comparison and clarified baseline variables associated with the incidence of SLE or RA. We found that BMI ≥24 kg/m^2^ was the only predictor of the low incidence of SLE or RA. This multicenter study reflected daily practice in the real world, and SVR to PR was not associated with the low incidence of SLE or RA in any subgroup analysis after adjustment for age, sex, and competing mortality.

Arthralgia is a common EM of HCV infection, and its prevalence is estimated up to 23%^2,7^. HCV-related arthritis could be polyarthritis involving small joints and intermittent mono-oligo-articular nondestructive arthritis involving large and medium joints, and the patterns of HCV-related arthritis are similar to those of mild RA presenting with polyarthritis^8^. The rheumatoid factor was identified in 50–80% of CHC patients^9^. The pathogenesis may be the direct invasion of synovial cells by HCV or a cytokine-induced disease^10^. Epidemiological studies have shown that the prevalence of HCV infection is significantly higher (10–11%) in SLE patients compared to the control group (1.0–1.3%)^11,12^, which suggests a potential interaction between HCV infection and SLE. Patients with SLE and CHC share many common immunological features, such as hypocomplementemia and the presence of antinuclear and anticardiolipin antibodies^13^. HCV does not have a DNA intermediate in its life cycle, and HCV RNA cannot integrate into the human genome. HCV may act as a chronic stimulus to the immune system^14,15^. The HCV envelope protein E2 can bind the CD81 molecule on the cell membrane of both hepatocytes and B-cells, and the binding on the B-cells may lower the threshold for the activation of B-cells^16,17^.

Hsu *et al.* reported that PR therapy does not decrease the incidence of catastrophic autoimmune disease^4^, and the present study revealed that SVR to PR does not decrease the incidence of SLE or RA. In this study, the observed incidence of SLE or RA was 21 per 67,930 person-years, which is similar to the incidence of 24.4 per 100,000 person-years (7.2, 95% CI: 6.5–8.0 in SLE; 17.2, 95% CI: 16.1–18.4 in RA) between 2005 and 2009 in Taiwan^18^. Why HCV eradication did not decrease the incidence of SLE or RA is yet to be studied, particularly in terms of immunological profiles before versus after HCV eradication.

The effect of BMI on the incidence of SLE or RA in CHC patients is still unknown. Escalante *et al.* showed that underweight patients with RA had higher mortality, which may be related to systemic inflammation^19^. SLE is a chronic inflammatory connective tissue disorder characterized by elevated proinflammatory cytokines in the blood, including leptin. Li *et al.* revealed that Asian SLE patients ≥ 40 years with a BMI < 25 had higher leptin^20^. We speculate that thinner HCV patients had higher systemic inflammatory cytokine levels, such as leptin, and the combination of systemic inflammatory cytokines and HCV may trigger SLE or RA in CHC patients.

MC is the most well-known EM in CHC patients, and 19–54% of CHC patients exhibit MC^21,22^. A systematic review revealed that the prevalence of MC vasculitis was 4.9% in CHC patients compared with 0% in non-HCV healthy people^22^. However, cryoglobulin measurement is not a routine practice in Taiwan due to the policy of Taiwan National Health Insurance and is usually not performed before SLE or RA diagnosis. Consequently, the events of MC were less than three in this study. SS is a systemic autoimmune disease (SAD) associated with HCV infection^23^ and has been diagnosed in 10–30% of CHC patients^22,24^. Due to less than three events, SS was not assessable in this cohort.

This study has several limitations. First, the events of several autoimmune diseases, including MC, chronic glomerulonephritis, autoimmune thyroiditis, lichen planus, SS, immune thrombocytopenic purpura, autoimmune hemolytic anemia, and porphyria cutanea tarda, were less than three; hence, these autoimmune diseases were not assessable. However, See *et al.* reported a low incidence of SS (11.8, 95% CI: 10.8–12.7) in Taiwan^18^, but the nationwide incidence of cryoglobulinemia was unknown. Second, we collected baseline virological features, complete blood count, and biochemical data, but the effect of baseline autoantibody profiles and their temporal changes on the incidence of autoimmune diseases remain unknown; therefore, further investigation is required. Finally, DAAs are currently the standard of care for CHC^5,6^, and whether HCV eradication by using DAAs exhibits a differential effect on the incidence of autoimmune diseases is yet to be elucidated.

In conclusion, CHC patients achieving SVR to PR therapy did not exhibit a low annual incidence of SLE or RA compared with non-SVR patients. Baseline BMI ≥ 24 kg/m^2^ was an independent predictor of the low incidence of SLE or RA in CHC patients.

## Materials and Methods

### Patients

T-COACH is a nationwide collaborative HCV registry cohort that includes 23 regional hospitals and medical centers of Taiwan. In total, 15,836 CHC patients who had received PR therapy for at least 4 weeks between January 2003 and December 2015 were enrolled. Taiwan Health Insurance administration has reimbursed PR therapy for CHC patients since 2003, and a total of 75,431 CHC patients were reimbursed for PR therapy between January 2003 and December 2015 (https://data.nhi.gov.tw/). The T-COACH consortium accounted for 21% of the treated CHC population in Taiwan over the 13-year period. The key inclusion criteria for the study were as follows: age ≥ 20 years, presence of the serum anti-HCV antibody for >6 months or compatible liver histology, detectable HCV RNA, and PR therapy for at least 4 weeks. Demographic data, virological features, complete blood count data, and biochemical data were collected at baseline, and therapeutic responses to PR therapy was also recorded. The exclusion criteria were liver diseases caused by other etiologies, hepatitis B virus coinfection, autoimmune diseases before undergoing PR therapy, and unavailable virological outcomes.

This study was conducted in accordance with the 1975 Declaration of Helsinki. All patients provided written informed consent prior to enrollment. This study was approved by the Research Ethics Committee of China Medical University Hospital, in Taichung, Taiwan (CMUH104-REC1-070) and each study site.

### Definition of autoimmune diseases

Patients with autoimmune diseases were identified on the basis of the specific codes of ICD-9-CM once at admission or on more than three occasions at the outpatient clinic by connecting to Taiwan NHIRD (Supplementary Table 1). However, only SLE (710.0) and RA (714.0) events were counted. Other autoimmune diseases, such as cryoglobulinemia (273.2), SS (710.2), and lichen planus (697.x), were not assessable because of few events (n <3) per NHIRD regulations.

### Laboratory tests

Complete blood count analyses, blood biochemistry tests, HCV RNA level determination, and HCV genotyping were performed in the central laboratory of each hospital. LC was defined by any of the following: liver histology^25^, transient elastography (FibroScan®; Echosens, Paris, France >12 kPa)^26^, acoustic radiation force impulse (>1.98 m/s)^27^, FIB-4 index (>6.5)^26^, and the presence of clinical, radiological, endoscopic, or laboratory evidence of cirrhosis.

### Statistical analyses

Continuous variables are reported as mean ± standard deviation (SD) and categorical variables as number (percentage). We considered death as a competing event, modified the Kaplan–Meier method according to Gray’s cumulative incidence method^28^, and compared the incidences of newly diagnosed SLE or RA between patients who achieved and those who did not achieve SVR. Subdistribution hazard models were used to estimate the HR and 95% CI for examining the independent factors associated with the major outcomes^29^. Subgroup analyses of stratified patients were performed to evaluate the SVR effect on the incidence of SLE or RA.

Statistical analyses were performed using the SAS Enterprise Guide (version 9.4, SAS Institute Inc., Cary, NC, USA) and a two-sided p value of <0.05 was considered statistically significant.

## Supplementary information

Supplementary information 

## References

[1] El-Serag HB, Hampel H, Yeh C, Rabeneck L (2002). Extrahepatic manifestations of hepatitis C among United States male veterans. Hepatology.

[2] Cacoub P (1999). Extrahepatic manifestations of chronic hepatitis C. MULTIVIRC Group. Multidepartment Virus C. Arthritis Rheum.

[3] Jacobson IM, Cacoub P, Dal Maso L, Harrison SA, Younossi ZM (2010). Manifestations of chronic hepatitis C virus infection beyond the liver. Clinical gastroenterology and hepatology: the official clinical practice journal of the American Gastroenterological Association.

[4] Hsu YC (2015). Association between antiviral treatment and extrahepatic outcomes in patients with hepatitis C virus infection. Gut.

[5] Panel A-IHG (2018). Hepatitis C Guidance 2018 Update: AASLD-IDSA Recommendations for Testing, Managing, and Treating Hepatitis C Virus Infection. Clin Infect Dis.

[6] European Association for the Study of the Liver. Electronic address, e. e. e. & European Association for the Study of the, L. EASL Recommendations on Treatment of Hepatitis C 2018. *Journal of hepatology* **69**, 461–511, 10.1016/j.jhep.2018.03.026 (2018). 10.1016/j.jhep.2018.03.026 29650333

[7] Buskila D (2000). Hepatitis C-associated arthritis. Curr Opin Rheumatol.

[8] Palazzi C, Olivieri I, Cacciatore P, Pennese E, D’Amico E (2005). Management of hepatitis C virus-related arthritis. Expert Opin Pharmacother.

[9] Lormeau C, Falgarone G, Roulot D, Boissier MC (2006). Rheumatologic manifestations of chronic hepatitis C infection. Joint Bone Spine.

[10] Dore MP, Fattovich G, Sepulveda AR, Realdi G (2007). Cryoglobulinemia related to hepatitis C virus infection. Dig. Dis. Sci..

[11] Ramos-Casals M (2000). Hepatitis C virus infection mimicking systemic lupus erythematosus: study of hepatitis C virus infection in a series of 134 Spanish patients with systemic lupus erythematosus. Arthritis and rheumatism.

[12] Ahmed MM (2006). Prevalence of active hepatitis C virus infection in patients with systemic lupus erythematosus. The American journal of the medical sciences.

[13] Manns MP, Rambusch EG (1999). Autoimmunity and extrahepatic manifestations in hepatitis C virus infection. J. Hepatol..

[14] Machida K (2006). Hepatitis C virus induces toll-like receptor 4 expression, leading to enhanced production of beta interferon and interleukin-6. J Virol.

[15] Peveling-Oberhag J, Arcaini L, Hansmann ML, Zeuzem S (2013). Hepatitis C-associated B-cell non-Hodgkin lymphomas. Epidemiology, molecular signature and clinical management. Journal of hepatology.

[16] Pileri P (1998). Binding of hepatitis C virus to CD81. Science.

[17] Dammacco F (2000). The lymphoid system in hepatitis C virus infection: autoimmunity, mixed cryoglobulinemia, and Overt B-cell malignancy. Seminars in liver disease.

[18] See LC, Kuo CF, Chou IJ, Chiou MJ, Yu KH (2013). Sex- and age-specific incidence of autoimmune rheumatic diseases in the Chinese population: a Taiwan population-based study. Semin Arthritis Rheum.

[19] Escalante A, Haas RW, del Rincon I (2005). Paradoxical effect of body mass index on survival in rheumatoid arthritis: role of comorbidity and systemic inflammation. Arch Intern Med.

[20] Li HM (2015). Plasma/Serum Leptin Levels in Patients with Systemic Lupus Erythematosus: A Meta-analysis. Arch. Med. Res..

[21] Landau DA (2010). Causes and predictive factors of mortality in a cohort of patients with hepatitis C virus-related cryoglobulinemic vasculitis treated with antiviral therapy. The Journal of rheumatology.

[22] Younossi Z, Park H, Henry L, Adeyemi A, Stepanova M (2016). Extrahepatic Manifestations of Hepatitis C: A Meta-analysis of Prevalence, Quality of Life, and Economic Burden. Gastroenterology.

[23] Ramos-Casals M (2005). Sjogren syndrome associated with hepatitis C virus: a multicenter analysis of 137 cases. Medicine (Baltimore).

[24] Cacoub P (2000). Extrahepatic manifestations associated with hepatitis C virus infection. A prospective multicenter study of 321 patients. The GERMIVIC. Groupe d’Etude et de Recherche en Medecine Interne et Maladies Infectieuses sur le Virus de l’Hepatite C. Medicine (Baltimore).

[25] Scheuer PJ (1991). Classification of chronic viral hepatitis: a need for reassessment. Journal of hepatology.

[26] Castera L (2005). Prospective comparison of transient elastography, Fibrotest, APRI, and liver biopsy for the assessment of fibrosis in chronic hepatitis C. Gastroenterology.

[27] Lin YH (2016). The performance of acoustic radiation force impulse imaging in predicting liver fibrosis in chronic liver diseases. The Kaohsiung journal of medical sciences.

[28] Gray RJ (1988). A Class of K-Sample Tests for Comparing the Cumulative Incidence of a Competing Risk. The Annals of Statistics.

[29] Fine JP, Gray RJ (1999). A Proportional Hazards Model for the Subdistribution of a Competing Risk. J. Am. Stat. Assoc..

