# Supplementary material for: Sustained virological response to hepatitis C therapy does not decrease the incidence of systemic lupus erythematosus or rheumatoid arthritis
Source: Sci Rep. 2020 Mar 25;10:5372. doi: 10.1038/s41598-020-61991-3 (PMC7096452; doi:10.1038/s41598-020-61991-3)
Supplement: Supplementary file 1 — Supplementary information [file 41598_2020_61991_MOESM1_ESM.docx]

**Sustained virological response to hepatitis C therapy does not decrease the incidence of systemic lupus erythematosus or rheumatoid arthritis**

Wei-Fan Hsu, Chi-Yi Chen, Kuo-Chih Tseng, Hsueh-Chou Lai, Hsing-Tao Kuo, Chao-Hung Hung, Shui-Yi Tung, Jing-Houng Wang, Jyh-Jou Chen, Pei-Lun Lee, Rong-Nan Chien, Chun-Yen Lin, Chi-Chieh Yang, Gin-Ho Lo, Chi‐Ming Tai, Chih-Wen Lin, Jia-Horng Kao, Chun-Jen Liu, Chen-Hua Liu, Sheng-Lei Yan, Ming-Jong Bair, Wei-Wen Su, Cheng-Hsin Chu, Chih-Jen Chen, Ching-Chu Lo, Pin-Nan Cheng, Yen-Cheng Chiu, Chia-Chi Wang, Jin-Shiung Cheng, Wei-Lun Tsai, Han-Chieh Lin, Yi-Hsiang Huang, Pei-Chien Tsai, Jee-Fu Huang, Chia-Yen Dai, Wan-Long Chuang, Ming-Lung Yu^*^ & Cheng-Yuan Peng^*^

**Supporting Information**

**Supplementary Table 1** ICD-9-CM codes for the diagnoses of autoimmune diseases

| Disease | Corresponding ICD-9-CM codes |
| --- | --- |
| Cryoglobulinemia | 273.2 |
| Chronic glomerulonephritis | 582.x |
| Autoimmune thyroiditis | 245.2 |
| Lichen planus | 697.x |
| Systemic lupus erythematosus | 710.0 |
| Rheumatoid arthritis | 714.0 |
| Sjögren’s syndrome | 710.2 |
| Immune thrombocytopenic purpura | 287.31 |
| Autoimmune hemolytic anemia | 283.0 |
| Porphyria cutanea tarda | 277.1 |

ICD-9-CM, International classification of Disease, Ninth revision, Clinical Modification
